# Efficacy of Repetitive Transcranial Magnetic Stimulation (rTMS) in the Treatment of Bulimia Nervosa (BN): A Review and Insight into Potential Mechanisms of Action

**DOI:** 10.3390/jcm13185364

**Published:** 2024-09-10

**Authors:** James Chmiel, Marta Stępień-Słodkowska

**Affiliations:** Faculty of Physical Culture and Health, Institute of Physical Culture Sciences, University of Szczecin, Al. Piastów 40B blok 6, 71-065 Szczecin, Poland

**Keywords:** rTMS, repetitive transcranial magnetic stimulation, bulimia nervosa, non-invasive brain stimulation, neurostimulation, neuromodulation, eating disorders

## Abstract

Introduction: Bulimia nervosa (BN) is a disorder primarily affecting adolescent females, characterized by episodes of binge eating followed by inappropriate compensatory behaviors aimed at preventing weight gain, including self-induced vomiting and the misuse of diuretics, laxatives, and insulin. The precise etiology of BN remains unknown, with factors such as genetics, biological influences, emotional disturbances, societal pressures, and other challenges contributing to its prevalence. First-line treatment typically includes pharmacotherapy, which has shown moderate effectiveness. Neuroimaging evidence suggests that altered brain activity may contribute to the development of BN, making interventions that directly target the brain extremely valuable. One such intervention is repetitive transcranial magnetic stimulation (rTMS), a non-invasive stimulation technique that has been garnering interest in the medical community for many years. Methods: This review explores the use of rTMS in the treatment of BN. Searches were conducted in the PubMed/Medline, ResearchGate, and Cochrane databases. Results: Twelve relevant studies were identified. Analysis of the results from these studies reveals promising findings, particularly regarding key parameters in the pathophysiology of BN. Several studies assessed the impact of rTMS on binge episodes. While some studies did not find significant reductions, most reported decreases in binge eating and purging behaviors, with some cases showing complete remission. Reductions in symptoms of depression and food cravings were also demonstrated. However, results regarding cognitive improvement were mixed. The discussion focused heavily on potential mechanisms of action, including neuromodulation of brain networks, induction of neuroplasticity, impact on serotonergic dysfunction, anti-inflammatory action, and HPA axis modulation. rTMS was found to be a safe intervention with no serious side effects. Conclusions: rTMS in the treatment of BN appears to be a promising intervention that alleviates some symptoms characteristic of the pathophysiology of this disorder. An additional effect is a significant reduction in depressive symptoms. However, despite these findings, further research is required to confirm its effectiveness and elucidate the mechanisms of action. It is also recommended to further investigate the potential mechanisms of action described in this review.

## 1. Introduction

The disorder known as bulimia nervosa (BN) that primarily affects adolescent females [1] is typified by binge eating and adverse compensatory actions intended to prevent weight gain [2]. Self-induced vomiting, abusing diuretics, abusing laxatives, and misusing insulin are some examples of such adverse behaviors [3]. The precise reason behind the emergence of BN is yet unknown. Numerous elements including genetics, biology, emotions, societal expectations, and other difficulties contribute to the rising prevalence of this eating disorder [4]. The perception of body weight is disturbed in people with BN. The majority of BN sufferers are either normal weight or only slightly overweight [5], and they frequently manage to hide their eating disorder from others [4]. Medical consequences that interfere with proper physiological functioning and raise the rates of morbidity and mortality are often associated with BN. Prolonged gastric acid reflux brought on by excessive vomiting might result in dysphagia and dyspepsia [6]. The excessive abuse of laxatives is a primary purging method in BN. Cathartic colon syndrome represents the last and most serious side effect of abusing laxatives [6].

There are various approaches to the treatment of BN. The initial line of treatment is psychological, such as cognitive behavioral therapy [7], although that is mediocrely effective [8]. Drug therapy is a further option for treatment. The most commonly prescribed medications are selective serotonin reuptake inhibitors (SSRIs), such as topiramate and fluoxetine [9]. The use of bupropion is decreasing [10]. Pharmacotherapy plus psychotherapy may have extra positive effects and these effects last over time [8]. Furthermore, the majority of research suggests that most people with BN seek treatment for weight loss rather than for their eating disorder [11].

Modern neuroimaging methods offer the possibility to understand the neural mechanisms of mental disorders such as BN. Numerous studies of functional magnetic resonance imaging and functional near-infrared spectroscopy indicate that BN is characterized by changes in brain activity at rest, during various paradigms, and in functional connectivity. Hypoactivity in the frontostriatal circuit along with altered striatal, orbitofrontal, insular and prefrontal cortical function are the most consistent observations [12], along with hyperactivity in the anterior cingulate cortex (ACC) and OFC [12]. Poor inhibitory control in the lateral prefrontal circuit also contributes to the tendency to binge eat [13]. There have also been reports of hypoactivation of executive control networks and hyperactivity of the parieto-occipital areas in BN patients [14].

As can be seen, BN is characterized by changes in the activity of many cortical regions. As such, therapeutic procedures that directly target these changes would be valuable. One treatment is repetitive transcranial magnetic stimulation (rTMS), a non-invasive brain stimulation technique that has been gaining great interest in the scientific community for years due to its non-invasiveness and effectiveness.

TMS generates an electromagnetic field in a wire coil. This field is then modulated over a specific area of the skull in predetermined pulsing patterns. By altering the excitability of the target population of neurons in the brain tissue underneath the coil’s center, the magnetic field pulses permeate the skull and help to either increase or decrease endogenous brain electrical activity [15]. This modulation can either enhance (facilitate) or inhibit (suppress) neuronal firing. The specific effects of rTMS depend on the parameters of the stimulation such as frequency, intensity and duration of treatment [16]. TMS can be supplied as a single pulse format, which can last just a fraction of a millisecond, or as paired pulses or repeated trains of pulses of tens of seconds to tens of minutes, depending on the desired outcome [17]. Repetitive TMS (rTMS) refers specifically to the repeated application of TMS pulses, which can sustain modulated brain activity over longer periods of time [18]. rTMS can be given in a single-session (one sitting) or in multi-sessions (several stimulation sessions spread over several days, usually consecutive) [19]. In laboratory research, single-session rTMS is the most common delivery format, while in clinical therapy studies, multi-session rTMS is the most common delivery approach [20]. When rTMS is applied, the motor cortex is typically targeted to create a customized cortical excitation threshold. This is not required, but it is a common practice. Single electromagnetic pulses of increasing intensity are applied until a consistent response criterion (such as a motor evoked potential or an observable finger twitch) is achieved [21]. The target region (such as the DLPFC) is subsequently localized. Typically, this is performed using an EEG cap in accordance with the International 10–20 system [22], or by employing structural MRI and neuronavigation tools/software [23].

rTMS has been studied and utilized for various neurological and psychiatric conditions. Potential applications of rTMS include treatment for anxiety disorders [24], post-traumatic stress disorder [25], and chronic pain conditions [26]. rTMS is a non-invasive method with a favorable safety profile. Common negative side effects include mild headaches, scalp discomfort, and transient dizziness [27]. Serious negative side effects, such as seizures, are rare and are generally associated with improper use or contraindications not being observed.

Several reviews have been published on the use of non-invasive brain stimulation in the treatment of eating disorders [28,29,30,31,32,33]. However, these reviews have several drawbacks. Primarily, the reviews combined several brain stimulation methods. This creates the risk of not capturing the effect of a single method, especially as each would work differently. Secondly, those reviews covered a broad spectrum of eating disorders that vary significantly in neurobiology, pathophysiology, symptoms and treatment approaches. Applying a specific therapeutic approach to anorexia nervosa may not be appropriate for the effective treatment of BN. Thirdly, not all available studies on the use of rTMS in BN were included, which limits evidence for the effectiveness of stimulation in BN. Finally, the potential mechanisms of action of rTMS in BN were not elucidated.

The purpose of this current review is to fill these gaps. As such, a comprehensive search for literature examining the use of rTMS in the treatment of BN was performed. All study results were analyzed in detail. The findings that look useful in future research and clinical applications were highlighted. Most importantly, the number of potential mechanisms of action of rTMS in BN were presented.

## 2. Methods

### 2.1. Data Sources and Search Strategy

J.Ch. and M.S.-S. carried out an independent internet search adhering to established standards while preparing this evaluation using the following set of combined keywords: “bulimia” OR “bulimia nervosa” OR “bulimic” AND “repetitive transcranial magnetic stimulation” OR “rTMS”. In July 2024 we made a comprehensive search across multiple databases such as PubMed/Medline, Research Gate and Cochrane, with a focus on articles from January 2000 to July 2024.

### 2.2. Study Selection Criteria

The papers had to be clinical trials and case studies in English published between 2000–2024, in order to meet the eligibility requirements for this study. It was necessary for this research to investigate, either as a primary or secondary result, how rTMS affected BN. All review papers and any not published in English were then excluded.

### 2.3. Screening Process

To ensure that the relevant research was included, and that any studies that did not meet the predefined criteria were rejected, many screening procedures were put in place. During the initial screening phase, J.Ch. and M.S.-S., two independent reviewers, carefully looked over the abstracts and titles.

#### 2.3.1. Title and Abstract Screening

Each reviewer evaluated the titles and abstracts of the records that were available on their own to determine which research fulfilled the inclusion criteria. At this point, the primary focus of the screening criteria was the effect and applicability of rTMS on BN.

#### 2.3.2. Full-Text Assessment

After titles and abstracts were first screened, the chosen papers were subjected to a thorough full-text assessment. The reviewers meticulously examined each article to ensure that it satisfied the eligibility requirements, with a particular focus on making sure the studies were clinical trials and case studies in English and published between January 2000 and July 2024.

## 3. Results

**Figure 1:**
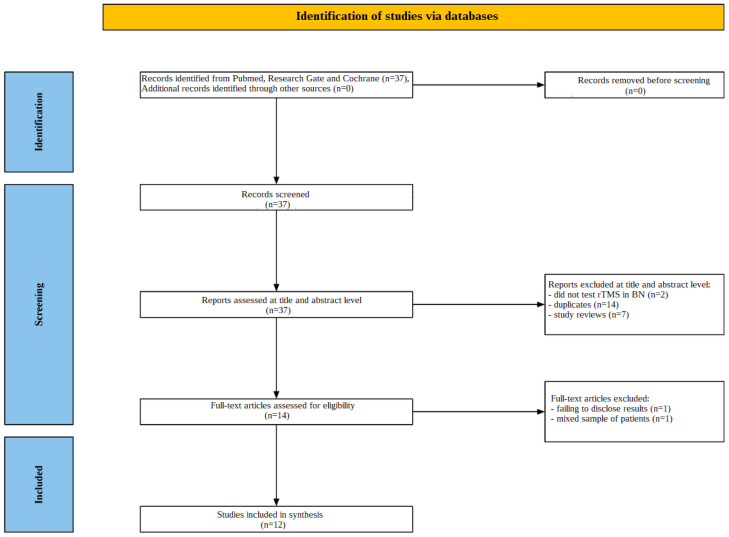
Flow chart depicting the different phases of the systematic review.

An illustration of the screening procedure can be found in Figure 1. Initially, search results across the many databases produced 37 studies. After examining the titles and abstracts of these studies, 23 were eliminated; two of them did not test rTMS in BN, 14 of them were duplicates, and seven of them were research reviews. A thorough full-text examination was then conducted on the 14 studies that were left. Two of these studies were not included. One was disqualified for failing to disclose results, while another was disqualified for having a mixed sample of patients with both BN and anorexia nervosa. Twelve articles were determined to fit the requirements for inclusion after a comprehensive study of the contents.

**Figure 2:**
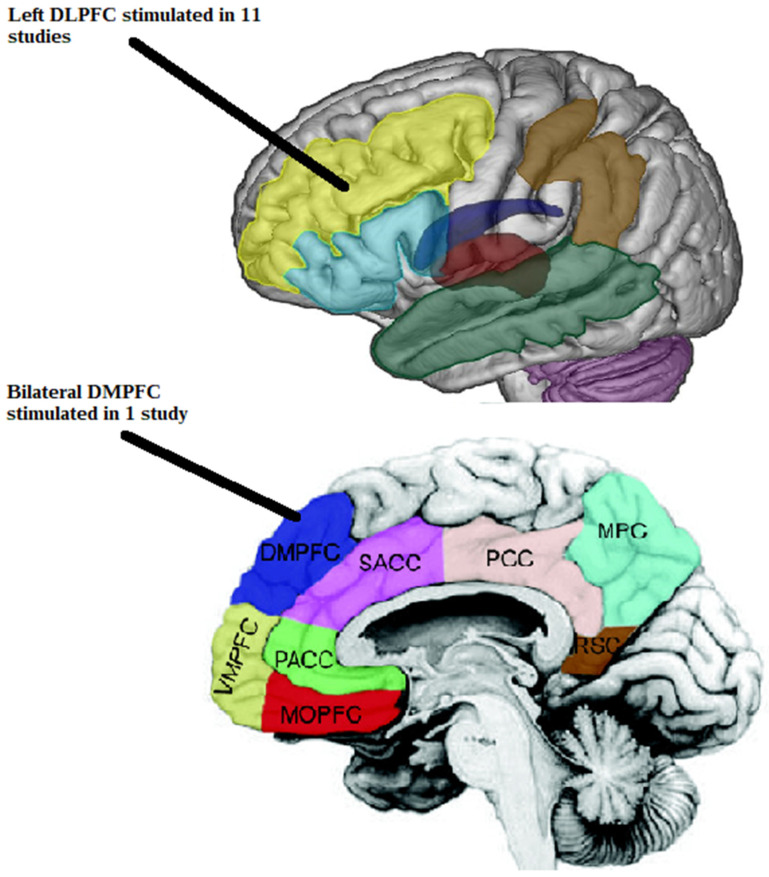
Cortical structures stimulated in the included studies. Left DLPFC stimulated in 11 studies: [34,35,36,37,38,39,40,41,42,43,45]. Bilateral DMPFC stimulated in 1 study: [44].

The studies found [34,35,36,37,38,39,40,41,42,43,44,45] were published between 2004 and 2018. Six studies were RCTs [34,35,37,39,40,42], three were pilot studies [36,38,41], and three were case studies [43,44,45]. Sham rTMS was a control group in seven studies [34,35,37,39,40,41,42], and in one study [38] the control group received active stimulation for comparison between left- and right-handed people. Three studies included only right-handed people [35,36,40], one study [38] involved left- and right-handed people, the remaining eight studies did not differentiate the participants [34,37,39,41,42,43,44,45]. Randomization occurred in seven studies [34,35,37,39,40,41,42], partial randomization occurred in one study [38], and no randomization occurred in four studies [36,43,44,45]. Six studies [34,35,37,39,40,42] were double-blind studies in which patients and assessors were blinded; three studies were unblinded [36,43,44]; two studies [38,41] were single-blind blinded where only patients were blinded; and one study [45] was single-blind where the assessor was blinded. All studies involved 262 participants (140 in the real rTMS group, 122 in the sham rTMS control group). Two stimulation sites were used in the included studies: the left DLPFC [34,35,36,37,38,39,40,41,42,43,45], and the bilateral DMPFC [44]. Figure 2 shows the cortical structures stimulated in the included studies.

### 3.1. Summary of Included Studies

The included studies are summarized in Table 1. Guillaume et al. [34] conducted a study to investigate how rTMS affects cognitive functions in individuals with BN. The research involved 39 participants, with 22 in the sham group and 17 in the real rTMS group. Over the course of 2 weeks, participants received either real rTMS (consisting of 10 sessions with 20 trains of 5 s each, separated by 55-s intervals, at a frequency of 10 Hz and an intensity of 110% of the motor threshold) or sham rTMS. The left dorsolateral prefrontal cortex (DLPFC) was stimulated. Cognitive assessments were carried out before the intervention and after the final rTMS session. The evaluation covered three dimensions: inhibitory control, assessed through a go/no-go task and the Barratt Impulsiveness Scale (BIS); decision-making, measured using the Iowa gambling task (IGT); and sustained attention, evaluated with the D2 test of attention.

Research [35] examined the therapeutic efficacy and safety of an adjunct high-frequency rTMS treatment that targets the left DLPFC. The 47 female patients were randomly assigned to the sham and real stimulation groups (real rTMS *n* = 23, sham rTMS *n* = 24). Ten rTMS sessions were conducted on the real group. A total of 1000 pulses were produced in 20 min every rTMS session, which comprised 20 trains of 5 s each, spaced 55 s apart, at a frequency of 10 Hz and a motor threshold intensity of 110%. The number of binge episodes in the 15 days following the final stimulation session was the primary outcome. Secondary outcomes were the number of vomiting episodes in the last 15 days and depression as measured by the Montgomery–Asberg Depression Rating Scale (MADRS). The mean MADRS depression score in the rear rTMS group was 11, indicating mild depression.

In [36] evaluated the effect of rTMS on short-term food cravings and other bulimic symptoms in patients with BN, as well as the change in cerebral oxygenation caused by the treatment. Eight women participated in this study. Near-infrared spectroscopy was used to examine changes in hemoglobin concentration in the left DLPFC during cognitive tasks that assessed self-regulatory control in response to food picture stimuli, both at baseline and following a single rTMS session. The left DLPFC was stimulated at a frequency of 10 Hz and an intensity of 110% of the motor threshold for each person. There were fifteen 5 s trains with a 55 s inter-train interval. Over the course of 20 min, 1000 pulses were delivered. The impact of the stimulation was evaluated using questionnaires on the symptoms of eating disorders (Eating Disorder Inventory-2, EDI-2, Eating Disorder Examination Questionnaire, EDEQ), the impact on anxiety and depression (Hospital Anxiety and Depression Scale, HADS), the impact on the severity of the disease (Clinical Global Impression of disease severity (CGI), impact on food craving (Food Craving Questionnaire-State Score, FCQ-SS) and impact on the Global Assessment of Functioning (GAF).

The investigation [37] was a double-blind randomized placebo-controlled trial conducted with 22 female subjects on the impact of rTMS on food cravings and salivary cortisol concentration levels. Eleven participants in each group were randomized to either the sham or real rTMS stimulation group. One session of high-frequency rTMS was used on the left DLPFC. A total of 1000 pulses were produced during a 20-min period by administering 20 trains of 5 s each, separated by 55-s intervals, at a frequency of 10 Hz and an intensity of 110% of the individual’s motor threshold. During the 90-min trial, salivary cortisol concentrations were measured four times: at the start of the experiment (T1), during the “food challenge task” (T2), just after administering rTMS (T3), and during the second “food challenge task” (T4). Food craving was measured using the Food Craving Questionnaire—State (FCQ-S) and 10-cm visual analogue scale (VAS) “urge to eat”. The probability of binge episodes, mood, and tension were measured (VAS).

Research [38] evaluated if rTMS would reduce craving in seven left-handed people with BN. Additionally, 14 right-handed people with BN received stimulation as a control condition. The left DLPFC in each patient received a single rTMS session at a frequency of 10 Hz and an intensity of 110% of the patient’s motor threshold, for 20 trains of 5 s with an intertrain interval of 55 s, totaling 1000 pulses during a 20-min period. The Food Craving Questionnaire-State (FCQ-S) score and five 10 cm Visual Analogue Scales (VAS) measuring “urge to eat”, “hunger”, “tension”, “mood” and “urge to binge-eat” (main outcome) were used as outcome measures before and after the rTMS session.

The trial in [39] involved 14 women with BN. Seven were allocated to the real rTMS group and seven to the sham rTMS group. For 3 weeks, 5 days a week, one session per day of stimulation at a frequency of 20 Hz and an intensity of 120% of the motor threshold was administered to the left DLPFC. In a session, 10 trains of 10 s each with a train interval of 60 s were run. In the actively treated group, patients received 2000 stimuli per session, for a total of 30,000 stimuli. Outcomes in the study measured the number of binge episodes and vomiting, as well as measures of depression (Hamilton Depression Rating Scale, HDRS) and the Beck Depression Inventory (BDI)). Compulsive symptoms were measured by the Yale-Brown Obsessive Compulsive Scale (YBOCS).

The experiment [40] involved 37 patients with BN with the aim of investigating the impact of a single rTMS session on cue-induced food cravings. Seventeen patients were assigned to a single session of real rTMS and 20 to a sham rTMS session. With a frequency of 10 Hz and an intensity of 110% of the patient’s motor threshold, 20 trains of 5 s each with intertrain intervals of 55 s were given, yielding a total of 1000 pulses during a 20-min period. The Food Craving Questionnaire-State (FCQ-S) score measuring food craving, a 10-cm visual analogue scale (VAS) measuring “urge to eat”, and VAS measuring “hunger”, “tension”, “mood” and “urge to binge eat” were among the outcome measures both before and after the particular rTMS session. Twenty-four hours following the evaluation, a follow-up contact was made to inquire about any binges that had happened since the rTMS (secondary outcome). In order to elicit a desire for food, participants saw a 2-min film consisting of four segments featuring people consuming appetizing dishes. Afterwards, they were shown a buffet containing the same items and asked to score the foods’ appearance, flavor and aroma. Lastly, the participants were instructed to finish the FCQ-S and rate the five VAS.

In the research [41] involved 33 patients with BN who were assigned to either the real rTMS group (*n* = 15) or the sham rTMS group (*n* = 18). The effect of a single rTMS session on selective attention, as measured by the Stroop color word task (SCWT), was investigated. A 10 Hz rTMS session was applied to the left DLPFC, 5 cm in front of the location of the maximum abductor pollicis brevis stimulation. A total of 20 5-s trains with inter-train intervals of 55 s were given at 110% of the subject’s motor threshold.

Study [42] checked whether rTMS used in patients with BN was safe for the heart. Thirty-eight patients were assigned to either the real rTMS (*n* = 18) or the sham rTMS (*n* = 20) group. One rTMS session was performed on the left DLPFC. A frequency of 10 Hz and an intensity of 110% of the motor threshold were used to administer 20 5-s trains with an intertrain interval of 55 s. The effects of the stimulation on blood pressure and heart rate were measured.

A single case study [43] detailed the effect of rTMS on a 27-year-old patient with BN and comorbid depression. rTMS was applied to the left DLPFC at a frequency of 25 Hz and an intensity of 110% motor threshold using a figure-of-eight coil. For 4 weeks, the patient had treatment 5 days a week, Monday through Friday. The 25 Hz stimulation was applied in 2-s trains with an interval of 25 s between each train. Twenty total treatments were administered, with each session consisting of 1000 pulses. Depression was measured using the Hamilton Depression Rating Scale (HDRS). Effects on binge eating and purging behaviors were also measured, but no outcome measures were reported.

In a single case study [44], the effect of rTMS on a 43-year-old female patient with severe refractory BN and comorbid depression was examined. She participated in 20 sessions (five sessions per week for 4 weeks) of neuro-navigated rTMS. Both right and left DMPFCs were each stimulated with 60 trains of 10 Hz stimulation at 120% of resting motor threshold in 5-s trains with a 10-s inter-train interval in each treatment session, for 3000 pulses to each hemisphere. Depression was measured by BDI-II and HDRS. The number of binge–purge episodes was also recorded.

In the third single case study [45], the effect of rTMS on a 28-year-old female patient with BN and depression was examined. Depression was measured using HDRS and BDI, and binge episodes were recorded in a Binge–Purge Diary. For 2 weeks (2 × 5 days), the left DLPFC was stimulated (10 trains × 10 s, 20 Hz, 80% motor threshold, 60-s train interval).

### 3.2. Effects on Attention

In paper [34], when looking at each group individually, the analysis showed a positive change in sustained attention performance after the rTMS sessions for both the real and sham groups (significant with *p* < 0.001 for each group). No exact results before and after were provided.

In study [41], no improvement in selective attention was noted in SCWT (card 1—outcomes before rTMS 55.5 and after 53, card 2 before 77.3 and after 77.8, interference before 22.3 and after 24.7).

### 3.3. Effects on Inhibitory Control

Work [34] showed a noteworthy enhancement in inhibitory control performance in the real rTMS group. This improvement was evident in both the number of commission errors on the go/no-go test (*p* = 0.01 in the real rTMS group and *p* = 0.3 in the sham group) and the BIS cognitive impulsivity subscale (*p* = 0.03 in the real rTMS group and 0.9 in the sham group).

### 3.4. Effects on Decision-Making

In study [34], there was no enhancement in the overall IGT 51–100 following rTMS. However, there was a marginally significant trend in the active rTMS group (*p* = 0.07). Notably, the intermediate score showed a significant improvement after rTMS (*p* = 0.002 for active rTMS, *p* = 0.12 in the sham group). No exact results before and after were provided.

### 3.5. Effects on Binge Episodes

In research [35], there was no significant reduction in the number of binge episodes in the real rTMS group (decrease from 10 to 7, *p* = 0.57) and in the sham rTMS group (decrease from 12.50 to 6, *p* = 0.49).

In study [37], the real rTMS group was less likely to have a binge episode than the sham rTMS group (however, no outcome measure was provided, *p* = 0.047).

In work [39], the number of binge episodes decreased in both groups (in the real rTMS group from 2.1 to 1.5, in the sham rTMS group from 2.9 to 2.1).

In the single case study [43], following the first week of rTMS treatment, there was a decrease in both depression symptoms and binge eating and purging behaviors. The patient reported two episodes of binge eating and one of purging in the first week, then four episodes of binge eating and two episodes of purging in the second week. After that, she had no further incidents of purging or binge eating. There was a complete remission of purging and binge eating episodes by the third week of rTMS.

In the single case study by Downar et al. [44], the patient documented in the daily journal a complete remission for the duration of the first week of treatment. She reported having one binge–purge episode each day in the second week. By session 11, they had once more resolved, and did not reoccur.

The single case study [45] demonstrated that after completing the treatment, the patient did not experience any binge and purge episodes. At baseline, the patient had an average of two binge episodes and vomited twice daily.

### 3.6. Effects on Vomiting Episodes

In study [35], there was no significant reduction in the number of vomiting episodes in the real rTMS group (decrease from 8.5 to 7, *p* = 0.07).

In study [39], the level of vomiting decreased in both groups, with the improvement greater in the active rTMS group (from 2.6 to 1.5).

### 3.7. Effects on Depression

In research [35], only the real rTMS group experienced a marginally significant drop in MADRS score from 11 to 5. A score of 5 indicates no depression.

In study [36], the HADS depression score improved, but it was not significant (from 8.8 to 8.6, *p* = 0.828). This score still indicated the presence of mild depression.

In paper [39], self-rated depression scores on the BDI improved in both groups (in real rTMS from 21.6 to 15.3). This indicates an improvement from moderate depression to mild depression. No significant change was reported in physician-rated depression (HDRS, no results provided).

In the single case study [43], from 29 at baseline, the patient’s HDRS score dropped to 21 after five sessions, 16 after 10, 11 after 15, and to 6 by the conclusion of 20 sessions. This indicates an improvement from very severe depression to no depression.

In the single case study by Downar et al. [44], after 11 sessions, the patient’s baseline BDI-II score, which was tracked daily, decreased from 28 to 7 and remained there. This indicates an improvement from moderate depression to minimal depression. Her baseline HDRS score was 26 (very severe depression), but by the end of treatment, her weekly score had reduced to 0.

In the single case study [45], HDRS depression scores decreased by 50% (from 20 to 10). This indicates an improvement from “severe depression” to “mild depression”.

### 3.8. Effects on Anxiety

In the study [36], a significant improvement in the HADS anxiety scores was observed (decrease from 12 to 10.20, *p* = 0.088).

### 3.9. Effects on Symptoms of Eating Disorders

In study [36], there were non-significant reductions in EDI-2 (from 111.20 to 102.6, *p* = 0.273) and EDEQ (from 5.54 to 5.52, *p* = 0.960).

Study [37] showed no effect of rTMS on tension (*p* = 0.107), mood (*p* = 0.175), hunger (*p* = 0.074) nor urge to binge-eat (*p* = 0.325) on the VAS scale.

In research [38], a worsening of mood in VAS scale was observed in left-handed people (from 5.3 to 3.2, *p* = 0.06). The remaining VAS parameters did not differ from before to after treatment.

In experiment [40], in the primary outcome of urge to eat in VAS, the real rTMS group showed a significant reduction (from 5.5 to 4.3). The VAS scales of hunger, tension, mood and urge to binge eat did not differ between the differently handed groups with both groups showing improvements.

### 3.10. Effects on the Severity of Disease

In study [36], there was no significant impact on CGI (from 4.60 to 4, *p* = 0.208).

### 3.11. Effects on Food Craving

In trial [36], no significant effect on FCQ-SS was observed (from 140.60 to 131.20, *p* = 0.076).

The study [37] showed a trend towards reducing food cravings was observed (the result after treatment was not provided, *p* = 0.056).

In work [38], a reduction in food craving was noted in the FCQ-S (from 45.1 to 34.9, *p* = 0.02).

In study [40], both groups showed improvement in FCQ-S, but in the real rTMS group it was greater (from 48.1 to 37.8).

### 3.12. Effects on Functioning

In study [36], a significant improvement in the GAF score was observed (from 57 to 59.4, *p* = 0.042).

### 3.13. Effects on Cortisol Concentration

In research [37], the real rTMS group had a significantly lower cortisol concentration at time points T3 and T4 (no numerical data on cortisol concentration were provided).

### 3.14. Effects on Obsessive–Compulsive Symptom

In study [39], both groups improved in YBOCS (in real rTMS from 25.6 to 17.3, in sham rTMS from 23.1 to 17.0).

### 3.15. Effects on Heart Parameters

In the work [42], the single rTMS session did not affect blood pressure or heart rate.

### 3.16. Follow-Ups

Study [40] measured the occurrence of binge episodes 24 h following the sessions. While 4 of 18 people in the sham rTMS group experienced a binge episode, none of the 16 people in the real rTMS group experienced a binge episode.

In the single case study [43], 12 weeks following the sessions, the response had remained consistent, as had the patient’s depressed symptoms, with a HAMD-17 score of 8.

The single case study [44] showed that after the final therapy session, the patient experienced complete remission of both depression and disordered eating for a duration of 64 days. The patient did experience an incident of binge eating and purging on days 65, 70 and 71 following treatment.

### 3.17. Neuroimaging Outcomes

In study [36], when comparing the initial condition of the left DLPFC to the condition 4 h after rTMS, the NIRS measurement showed a substantial decrease in the left prefrontal [oxyHb] activation for the neutral photo stimuli. The latter part of the photo task period saw this [oxyHb] decline. There was no discernible difference in the prefrontal activations pre-rTMS and post-rTMS for any of the two food stimuli. For each measurement occasion, there was no discernible variation in the [oxyHb] activations between the three picture sets.

### 3.18. Safety

rTMS was found to be a safe intervention in the included studies, but few studies reported any information about side effects. The most common headaches were transient.

## 4. Discussion

Bulimia nervosa (BN) is a severe eating disorder that has a devastating impact on the individual’s quality of life and daily functioning. The included studies in this review provide a comprehensive examination of the effects of repetitive transcranial magnetic stimulation (rTMS) on various aspects of BN. Guillaume et al. [34] focused on cognitive functions, particularly inhibitory control, decision-making, and sustained attention in individuals with BN. The study involved 39 participants receiving either real or sham rTMS. A similar study [35] evaluated the therapeutic efficacy and safety of high-frequency rTMS targeting the left DLPFC in 47 female bulimics. Study [36] looked at the impact of rTMS on short-term food cravings and other bulimic symptoms in eight women. Study [37] investigated the effects of rTMS on food cravings and salivary cortisol levels with 22 female subjects. Study [38] explored the impact of rTMS on craving in left-handed versus right-handed individuals with BN. Study [39] examined the long-term effects of rTMS on binge episodes and depression in 14 women over 3 weeks. Study [40] aimed to determine the impact of a single rTMS session on cue-induced food cravings in 37 patients. Study [41] assessed the effect of rTMS on selective attention in 33 patients with BN. Study [42] evaluated the cardiovascular safety of rTMS in 38 patients. Lastly, three single case studies [43,44,45] investigated the effects of rTMS on individual patients with BN and comorbid depression.

Analysis of the results from the included studies provides some promising findings, in particular around key parameters in the pathophysiology of BN. Studies [35,37,39,43,44,45] assessed the impact of rTMS on binge episodes. Two of the studies [35,39] found non-significant reductions, while four others [37,43,44,45] reported a decrease in binge eating and purging behaviors, with some cases showing complete remission. In study [35], the number of binge episodes in the 15 days before treatment was 10 (0.67 episodes per day), while after treatment, it had reduced to 7 (0.46 episodes per day). Likewise, in study [39], the initial number of binge episodes was 2.1 per day, and after treatment 1.5 per day. In clinical trial [37], that showed positive results, participants had an average of 0.6 binge eating episodes per day before treatment. Significant improvement was demonstrated, but unfortunately not quantified. Therefore, it is not possible to compare the results with other studies. However, the results from these three studies and three case studies show that rTMS may be considered a promising method for reducing binge eating episodes in BN. Further studies in larger numbers of patients are needed to confirm these findings.

Two research [35,39] investigated the effect of rTMS on the number of vomiting episodes. Study [35] found a non-significant reduction in vomiting episodes. Study [39] also noted an improvement in the vomiting index in the active rTMS group. Before treatment, people in study [35] had an average of 8.5 episodes of vomiting in the prior 15 days (0.56 episodes per day), and after treatment there were 7 episodes in the following 15 days (0.47 episodes per day). In a study with positive results [39], people with BN had 2.6 episodes of vomiting per day before treatment, and after treatment 1.5 episodes of vomiting per day. It follows that multi-session rTMS may be an effective tool in reducing the frequency of vomiting in BN when the frequency of vomiting episodes is higher—then the improvement is more noticeable. Surprisingly, although forced vomiting is a core symptom of BN, only two of the 12 studies included this measure in the review (discussed in the penultimate section of this article).

Studies [35,36,39,43,44,45] examined the effects of rTMS on depression. While one study [36] found marginal or no improvements, others [35,39,43,44,45] reported significant reductions in depression scores, indicating a move from severe to mild or no depression. The reason for the lack of results in study [36] may be that the single rTMS session may have been insufficient to produce a measurable effect (evidence for this is provided in the next section on the mechanisms of action of rTMS).

Articles [36,37,38,40] assessed the impact of rTMS on food cravings. While one study [36] found no significant effects, others [37,38,40] reported reductions in food craving scores. The reason for the lack of effect in study [36] may again be the fact that only a single session was used. Results from the other three studies show that multisession rTMS may be effective in reducing food cravings in patients with BN.

rTMS has mixed effects on cognitive function in people with BN. As for attention, in study [34], rTMS improved sustained attention in both the sham and real rTMS groups. In study [41], rTMS did not improve selective attention. It is worth noting that this study used a single session, which may not have been sufficient to produce a noticeable effect. Together, both results do not indicate a positive effect of rTMS on attention in people with BN, and further research is necessary to demonstrate this effect. As for control inhibitors, study [34] showed a beneficial effect on this parameter. Even though improvement occurred in both groups, it was greater in the active rTMS group. A similar result was shown in the same study, but in decision-making. Further studies are required to demonstrate the effect of rTMS on control and decision-making inhibitors in patients with BN.

Positive results have been demonstrated in terms of the effect of rTMS on anxiety symptoms in patients with BN. Study [36] reported a significant reduction in anxiety symptoms following rTMS treatment. The average HADS-A score decreased from 12 to 10.20, indicating a move from moderate to mild anxiety. This suggests that rTMS can effectively reduce anxiety symptoms in patients who initially present with moderate levels of anxiety.

The effects of rTMS on the general symptoms of eating disorders in individuals with BN were evaluated in several studies, but produced mixed results. In study [36], the impact of rTMS on eating disorder symptoms was assessed using two standardized questionnaires: the Eating Disorder Inventory-2 (EDI-2) and the Eating Disorder Examination Questionnaire (EDEQ). There was a non-significant improvement in the EDI-2 scores, with an average score decreasing from 111.20 to 102.6. Although this reduction suggests a trend towards improvement, the change was not statistically significant, indicating that rTMS did not have a strong effect on the broader symptoms of eating disorders as measured by the EDI-2. Similarly, the EDEQ scores showed a minimal decrease from 5.54 to 5.52. This negligible change suggests that rTMS did not substantially impact the eating disorder behaviors and attitudes measured by the EDEQ in this study. This may be because this study used a single rTMS stimulation, which may be insufficient to affect a broad spectrum of eating disorder symptoms. 

Paper [38] differentiated between left-handed and right-handed individuals with BN to evaluate the effects of a single rTMS session on mood and eating disorder symptoms. A worsening of mood was observed in left-handed participants post-treatment. For the right-handed group, there were no significant differences in VAS parameters measuring mood, urge to eat, hunger, tension or urge to binge eat, for both before and after the rTMS session. This indicates that a single rTMS session may have a varied impact on mood based on handedness, but does not significantly alter the core symptoms of eating disorders in the short term. Similar to study [36], a single rTMS session may have been insufficient to influence the broad pathology of eating disorders. Study [40] also measured the impact on VAS for various parameters of eating disorders and showed that a single session affects the urge to eat. The effect on other parameters was also positive, but they also occurred in the sham stimulation group. The results from these three studies indicate that further studies, preferably examining the effects of multiple sessions, are needed to demonstrate the effect of rTMS on the overall pathology of eating disorders in BN.

Study [36] evaluated the impact of rTMS on the severity of BN using the CGI-S scale. The results indicated a slight but notable reduction in CGI-S scores following rTMS treatment. The average CGI-S score decreased from 4.6 (moderately ill) to 4.0 (moderately ill), suggesting a modest improvement in the overall severity of the disease. There may be two reasons for this. First, while the CGI-S scale provides a broad measure of severity of illness, it might not be sensitive enough to detect subtle improvements in specific symptoms or domains affected by rTMS. Additional specific measures could provide more detailed insights into the changes induced by rTMS. Second, a single rTMS stimulation session was used in this study and may not be sufficient to change disease severity scores. Further research is needed to demonstrate the effect of rTMS on CGI performance, preferably using multiple rTMS sessions.

The effects of rTMS on the overall functioning of individuals with BN were assessed in study [36] using the Global Assessment of Functioning (GAF) scale. The GAF scale is a numeric scale (0 through 100) used by mental health clinicians to rate the social, occupational and psychological functioning of adults. Higher scores indicate better functioning. The results indicated a modest but statistically significant improvement in GAF scores following rTMS treatment. The baseline average GAF score of 57 indicated moderate symptoms or moderate difficulties. After the rTMS treatment, the average GAF score rose to 59.4, still within the range indicating moderate difficulty but closer to the threshold for mild symptoms or some difficulty in functioning. Further research is required to confirm this effect.

In research [37], rTMS reduced cortisol levels. This proves the ability of rTMS to reduce the activity of the HPA axis. This effect is discussed in more detail in the next section.

In study [39], rTMS improved obsessive–compulsive symptoms scores on the YBOCS. This is consistent with the results of the meta-analysis [46] according to which rTMS applied to the left DLPFC area reduces OCD symptoms.

Study [42] showed that a single rTMS session did not affect cardiac parameters in patients with BN. This proves the neutral effect of stimulation on the heart. A meta-analysis [47] from 2023 showed that excitatory rTMS can improve cardiac measures (reduce blood pressure, improve heart rate variability). Therefore, rTMS can be used in patients with BN and cardiac dysfunction without fear of negative effects.

Reference [38] drew attention to an important issue in rTMS research—that left-handed people are often excluded from studies. This means that we do not know the effect of stimulation on left-handed people. In this study, rTMS worsened mood on the VAS scale in left-handed people and improved it in right-handed people. There was no effect on other VAS indicators. In both groups, rTMS reduced food cravings. This study opens an interesting area of research, but these results should be treated with caution, because the impact of a single rTMS session was examined, and the group of left-handed people tested was small (7 people). A 2021 meta-analysis [48] indicates that left-handed people respond as well to the treatment of depression by stimulating the left DLPFC as do right-handed people.

The follow-up assessments indicate that rTMS may have lasting effects on reducing binge eating and purging behaviors and on depression in some individuals with BN. The protective effect of rTMS appears to be sustained for several weeks to even months post-treatment, as seen in the case studies [43,44]. However, there is also evidence of some symptom recurrence in some cases, suggesting that the benefits of rTMS might diminish over time without continued intervention. The variability in follow-up outcomes highlights the need for larger-scale studies with extended follow-up periods to better understand the long-term efficacy and potential need for maintaining rTMS treatments in BN.

## 5. Potential Mechanisms of Action of rTMS in BN

Although little is known about the possible mechanisms of action of rTMS in BN, as only one neuroimaging study has been performed and various biomarkers (e.g., from blood) have not been used, it is still possible to discuss the possible mechanisms of action of rTMS. To achieve this, it is necessary to analyze the general mechanisms of action of modulated electromagnetic stimulation in other applications. Below are some areas where rTMS may work to treat BN.

### 5.1. Neuromodulation of Brain Networks

Findings from studies of both healthy [49] and ill individuals [50] indicate that frontostriatal circuits underlie the capacity for self-regulatory control. BN is associated with deficits in self-regulatory control, encompassing executive control, emotional regulation, and the ability to delay gratification [51]. MRI findings suggest that these circuits are structurally [52] and functionally [53] abnormal in BN, likely contributing to the loss of control over eating behaviors characteristic of the disorder [54]. Mounting evidence shows that abnormalities in frontostriatal circuitry in BN contribute to deficits in cognitive control, self-regulation of emotional and motor responses, and goal-directed behavior [55]. For example, BN patients exhibit hypoactivity and deficient engagement in frontostriatal circuitry during cognitive control tasks, like in the Simon Spatial Incompatibility task [56,57,58] and the go/no-go task [59], both in adults and adolescents. Healthy individuals activate regions within frontostriatal circuits for correct responses to incongruent stimuli [57,58]. In contrast, adults with BN show reduced activation in bilateral inferior frontal gyrus (IFG), dorsal striatum and anterior cingulate cortex (ACC) during correct responses to incongruent stimuli, with greater reductions in more severe BN cases [57]. Adolescents with BN also exhibit reduced conflict-related activation in these regions during conflict resolution, particularly during maximal conflict resolution [58].

In the study by Dunlop et al. [60], rTMS was used on the left dmPFC in patients with anorexia nervosa and BN. The aim was to identify predictors of responses to rTMS treatment. Using seed-based functional connectivity (FC) and the neighboring dorsal anterior cingulate cortex (dACC) and dmPFC as regions of interest, neural predictors and correlates of responses were found. Baseline FC from dmPFC to right posterior insula and lateral orbitofrontal cortex, as well as from dACC to right posterior insula and hippocampus, was lower in treatment responders. Responder FC between the anterior insula and ventral striatum and the dACC was modest at baseline, but it grew with therapy. Frontostriatal FC was elevated in nonresponders at baseline, and dmPFC-rTMS decreased FC in correlation with a worsening of symptoms. The study’s findings that treatment responders exhibited changes in frontostriatal connectivity align with the notion that these circuits are pivotal for self-regulation. The increased connectivity in responders could be indicative of improved functional integration in these circuits, potentially leading to better cognitive and emotional control, as well as a reduction in binge/purge behaviors. On the other hand, the paradoxical suppression of frontostriatal connectivity in nonresponders, associated with symptomatic worsening, underscores the complexity of BN and suggests that simply modulating connectivity may not be uniformly beneficial. This complexity mirrors the nuanced deficits in self-regulatory control. Research claims that stimulation of the prefrontal cortex, which is a large area of the brain, includes both the DLPFC and other prefrontal areas nearby [61]. It can be conservatively assumed that similar results would be obtained with stimulation of the left DLPFC, which was the case in most of the studies included in this review.

### 5.2. rTMS Can Induce Neuroplasticity in BN

Neuroplasticity is the brain’s ability to reorganize itself by forming new neural connections throughout life. This remarkable capability allows the neurons in the brain to compensate for injury and disease, and to adjust their activities in response to new situations or changes in the environment. Nonadaptive neuroplasticity refers to the brain’s ability to reorganize itself in a manner that is not beneficial and may even be detrimental. This type of neuroplasticity can occur in response to injury, disease or maladaptive behavior patterns, leading to negative outcomes such as maladaptive learning or the perpetuation of mental health disorders. In the context of BN, where we are dealing with learned harmful behaviors, it can be said that this disorder is also characterized by nonadaptive neuroplasticity. There are many biomarkers of neuroplasticity. The most famous is brain-derived neurotrophic factor (BDNF). BDNF is a crucial molecule contributing to neuronal plasticity and the binding processes that underlie long-term potentiation, learning and memory [62]. BDNF expression changes are linked to psychiatric disorders and both pathological and normal aging, especially in memory-related structures such as the hippocampus and parahippocampal regions [63]. Moreover, BDNF influences neurotransmitter networks, including those involving dopamine [64,65], serotonin [66] and glutamate [67]. In addition to its activity as a neurotrophic factor, BDNF also modulates and integrates signaling pathways in the immunological, endocrine and neurological systems [68]. It also affects the metabolic, eating and weight regulation processes [69]. In studies using animal models, BDNF was found in brain regions that are known to affect eating, including the paraventricular nucleus (PVN), dorsal vagal complex (DVC) and hypothalamus [70,71]. Animal models have demonstrated the involvement of BDNF and the TrkB receptor in eating and weight issues, with the absence of either protein linked to obesity and hyperphagia [72,73]. It has been shown that in BN the level of BDNF in the blood is reduced [74,75]. As for the direct mechanism through which reduced BDNF levels cause eating habits to be disrupted, it has been proposed that in binge eating disorder, a frequent form of BN, susceptibility is increased by single nucleotide polymorphisms (SNPs) in the BDNF gene, and that individuals with EDs have elevated levels of genetically modified BDNF molecules. These functional polymorphisms are frequently linked to decreased BDNF levels in the blood [74]. The therapeutic advantages of rTMS are thought to be mediated by BDNF [76], and the alterations in BDNF that have been reported in peripheral blood may be the result of rTMS-induced modulation of BDNF-TrkB signaling in the brain [77,78]. rTMS can help the brain adapt and heal by triggering a neuroplasticity mechanism through the synthesis of BDNF, which helps to alleviate BN symptoms. The effect of increasing BDNF levels in BN may be valuable, as successful treatment of BN has been shown to be associated with an increase in BDNF levels [79]. Moreover, in BN there is a disorder of the serotonin (5-HT) system (written about in another section) [80,81]. The regulation of food intake by BDNF has been closely linked to the brain’s 5-HT systems, indicating that endogenous BDNF is essential for the proper growth and operation of central 5-HT neurons [82]. Therefore, the modulatory effect of rTMS on BDNF may indirectly affect the function of the serotonergic system in BN.

### 5.3. Impact of rTMS on Serotoninergic Dysfunction in BN

In BN, there is disturbed secretion of neurotransmitters, including serotonin (5-HT) [1,2,3,4,5,6,7,8,9,10,11,12,13,14,15,16,17,18,19,20,21,22,23,24,25,26,27,28,29,30,31,32,33,34,35,36,37,38,39,40,41,42,43,44,45,46,47,48,49,50,51,52,53,54,55,56,57,58,59,60,61,62,63,64,65,66,67,68,69,70,71,72,73,74,75,76,77,78,79,80,81,82,83]. In developing humans, serotonin controls cell division, migration and proliferation [2,3,4,5,6,7,8,9,10,11,12,13,14,15,16,17,18,19,20,21,22,23,24,25,26,27,28,29,30,31,32,33,34,35,36,37,38,39,40,41,42,43,44,45,46,47,48,49,50,51,52,53,54,55,56,57,58,59,60,61,62,63,64,65,66,67,68,69,70,71,72,73,74,75,76,77,78,79,80,81,82,83,84]. In mature humans, it controls emotion, cognition, sleep, appetite, pain, circadian rhythms and endocrine function [3,4,5,6,7,8,9,10,11,12,13,14,15,16,17,18,19,20,21,22,23,24,25,26,27,28,29,30,31,32,33,34,35,36,37,38,39,40,41,42,43,44,45,46,47,48,49,50,51,52,53,54,55,56,57,58,59,60,61,62,63,64,65,66,67,68,69,70,71,72,73,74,75,76,77,78,79,80,81,82,83,84,85]. The 5-HT transporter is responsible for the reuptake of extracellular 5-HT, which regulates the amount and duration of serotonergic neurotransmission. 5-HT is synthesized from tryptophan and is subject to the rate-limiting regulation of tryptophan hydroxylase (TPH). 5-HT increases feelings of satiety and decreases the intake of protein and carbohydrates through its activity in the medial and lateral hypothalamus [4,5,6,7,8,9,10,11,12,13,14,15,16,17,18,19,20,21,22,23,24,25,26,27,28,29,30,31,32,33,34,35,36,37,38,39,40,41,42,43,44,45,46,47,48,49,50,51,52,53,54,55,56,57,58,59,60,61,62,63,64,65,66,67,68,69,70,71,72,73,74,75,76,77,78,79,80,81,82,83,84,85,86]. Appetite dysregulation and dyscontrol [5,6,7,8,9,10,11,12,13,14,15,16,17,18,19,20,21,22,23,24,25,26,27,28,29,30,31,32,33,34,35,36,37,38,39,40,41,42,43,44,45,46,47,48,49,50,51,52,53,54,55,56,57,58,59,60,61,62,63,64,65,66,67,68,69,70,71,72,73,74,75,76,77,78,79,80,81,82,83,84,85,86,87,88] may all be influenced by 5-HT abnormalities in BN. Moreover, some of the behavioral-trait manifestations like anxious and obsessional behaviors, extremes of impulse control, as observed in individuals with bulimic disorders, are similarly moderated by changes in 5-HT activity [7,8,9,10,11,12,13,14,15,16,17,18,19,20,21,22,23,24,25,26,27,28,29,30,31,32,33,34,35,36,37,38,39,40,41,42,43,44,45,46,47,48,49,50,51,52,53,54,55,56,57,58,59,60,61,62,63,64,65,66,67,68,69,70,71,72,73,74,75,76,77,78,79,80,81,82,83,84,85,86,87,88,89,90]. A large body of research indicates that disruptions in monoamine function happen in BN patients and continue even after they recover from BN [1,2,3,4,5,6,7,8,9,10,11,12,13,14,15,16,17,18,19,20,21,22,23,24,25,26,27,28,29,30,31,32,33,34,35,36,37,38,39,40,41,42,43,44,45,46,47,48,49,50,51,52,53,54,55,56,57,58,59,60,61,62,63,64,65,66,67,68,69,70,71,72,73,74,75,76,77,78,79,80,81,82,83,84,85,86,87,88,89,90,91,92,93]. Brain-imaging techniques reveal abnormal 5-HT function in both actively ill and recovered patients. Available data show decreased binding of the 5HT2a receptor in subgenual cingulate, mesial temporal, and parietal cortical regions in women who have recovered from BN [12,13,14,15,16,17,18,19,20,21,22,23,24,25,26,27,28,29,30,31,32,33,34,35,36,37,38,39,40,41,42,43,44,45,46,47,48,49,50,51,52,53,54,55,56,57,58,59,60,61,62,63,64,65,66,67,68,69,70,71,72,73,74,75,76,77,78,79,80,81,82,83,84,85,86,87,88,89,90,91,92,93,94], increased activity of presynaptic 5-HT1A autoreceptor in the dorsal raphe in individuals who have recovered from bulimic-type AN; and in postsynaptic 5HT1A receptors in different brain regions in individuals with active BN [13,14,15,16,17,18,19,20,21,22,23,24,25,26,27,28,29,30,31,32,33,34,35,36,37,38,39,40,41,42,43,44,45,46,47,48,49,50,51,52,53,54,55,56,57,58,59,60,61,62,63,64,65,66,67,68,69,70,71,72,73,74,75,76,77,78,79,80,81,82,83,84,85,86,87,88,89,90,91,92,93,94,95], decreased availability of the central 5-HT transporter in women with BN [14,15,16,17,18,19,20,21,22,23,24,25,26,27,28,29,30,31,32,33,34,35,36,37,38,39,40,41,42,43,44,45,46,47,48,49,50,51,52,53,54,55,56,57,58,59,60,61,62,63,64,65,66,67,68,69,70,71,72,73,74,75,76,77,78,79,80,81,82,83,84,85,86,87,88,89,90,91,92,93,94,95,96] or those who have recovered from bulimic anorexia nervosa [4,5,6,7,8,9,10,11,12,13,14,15,16,17,18,19,20,21,22,23,24,25,26,27,28,29,30,31,32,33,34,35,36,37,38,39,40,41,42,43,44,45,46,47,48,49,50,51,52,53,54,55,56,57,58,59,60,61,62,63,64,65,66,67,68,69,70,71,72,73,74,75,76,77,78,79,80,81,82,83,84,85,86,87,88,89,90,91,92,93,94,95,96]. Several studies have shown that rTMS applied to the left DLPFC modulates serotonergic activity [16,17,18,19,20,21,22,23,24,25,26,27,28,29,30,31,32,33,34,35,36,37,38,39,40,41,42,43,44,45,46,47,48,49,50,51,52,53,54,55,56,57,58,59,60,61,62,63,64,65,66,67,68,69,70,71,72,73,74,75,76,77,78,79,80,81,82,83,84,85,86,87,88,89,90,91,92,93,94,95,96,97,98]. Changes in serotonin release were shown to be connected with improvements in symptoms in the included studies, indicating that serotonin release and metabolism may be modulated in relation to the therapeutic benefits of HF-rTMS [18,19,20,21,22,23,24,25,26,27,28,29,30,31,32,33,34,35,36,37,38,39,40,41,42,43,44,45,46,47,48,49,50,51,52,53,54,55,56,57,58,59,60,61,62,63,64,65,66,67,68,69,70,71,72,73,74,75,76,77,78,79,80,81,82,83,84,85,86,87,88,89,90,91,92,93,94,95,96,97,98,99]. In the context of BN, serotonin modulation via rTMS may influence appetite control and reduction, and reduce obsessive and impulsive behaviors. Ultimately, it may result in a reduction in vomiting and purging behavior.

### 5.4. Anti-Inflammatory Action of rTMS

Neuroinflammation is a phenomenon that involves activation of the brain’s immune system. This response is mediated primarily by microglia, brain immune cells and astrocytes, which create an inflammatory environment. Astrocytes play a crucial role in maintaining neuronal balance. They offer metabolic support to neurons, help clear metabolic waste via the glymphatic system, reduce excitotoxicity by removing glutamate from synapses, regulate blood flow, control potassium levels, aid in synapse formation and communication, and manage neuroinflammation [100]. Microglia are involved in the central nervous system’s immune defense, and they also play key roles in regulating neurogenesis, neuroinflammation, synaptic pruning and synaptic plasticity [100]. Neuroinflammation occurs in many neurological and psychiatric conditions. Chronic high-fat food intake is known to cause both peripheral and central inflammation, which is linked to weight gain and comorbidities associated with obesity, such as type 2 diabetes, metabolic syndrome and insulin resistance [101]. Proinflammatory cytokines, such as TNF-α, IL-1β and IL-6, can directly affect the neural circuits in the hypothalamus that regulate appetite [102]. Patients with BN have been shown to have elevated levels of pro-inflammatory cytokines, including IL-1β, IL-6 and TNF-α. [103,104,105]. rTMS reduces the level of pro-inflammatory cytokines and regulates microglial function [106], which has been described in pathological conditions such as depression [107] and neuropathic pain [108]. rTMS also reduced astrocytic reactivity, which results in reduced neuroinflammation [100]. Reducing neuroinflammation in BN through rTMS may help regulate appetite.

The role of neuroinflammation in mediating cognitive decline, for example in depression, and in mediating decision-making and impulsivity in general is known. People suffering from BN have impaired decision-making [109]. The dysfunction of this cognitive construct is complex, but may be related to neuroinflammation [110,111]. The anti-inflammatory effects of rTMS in BN may improve decision-making, which may result in a reduced tendency to engage in purging behavior and induced vomiting. Another cognitive construct damaged in BN is impulsivity [112]. Some research suggests that neuroinflammation causes increased impulsivity, or that increased impulsivity causes neuroinflammation [113]. Regardless of which mechanism is true (or perhaps both are), lowering neuroinflammation via rTMS in BN patients may lower impulsivity, which may result in lower frequency of purging and compulsive eating behaviors.

Interestingly, the rTMS did not exhibit these anti-inflammatory effects in healthy subjects, indicating that when the inflammatory balance is upset, it may lessen an inappropriate excess of inflammatory and pro-inflammatory mediators and enhance anti-inflammatory factors to support the restoration of homeostasis [114,115,116].

### 5.5. HPA Axis Modulation

Stress plays a significant role in the onset and maintenance of BN [117]. The key control regions of the stress system are located in the brain stem and hypothalamus. A crucial component of this system is the hypothalamic-pituitary-adrenal (HPA) axis, which regulates hormone balance in response to stress and involves both the endocrine and central neurological systems. HPA axis reactivity has been hypothesized as one of the biological mechanisms linked to eating behavior [118]. Cortisol, a steroid hormone, is the end product of HPA axis activation, occurring in response to both physical and psychosocial stressors [119]. Given the physiological stress associated with food restriction [120] and the importance of stressful life events in the initiation [121,122] and maintenance [123] of eating disorders, cortisol has been a focal point in hormone studies related to eating disorders. Several studies have shown that individuals with BN have elevated cortisol levels [124,125,126]. Early research suggested that recurrent episodes of excessive food consumption might contribute to these observed effects; however, the mechanisms behind cortisol changes in BN are not fully understood [125]. Both the physiological sensations caused by consuming large meals and the psychological stress they generate activate the HPA axis. These factors facilitate the hyperactivation of the HPA axis, evidenced by elevated cortisol production and a hyporeactive adrenocorticotropin response to corticotrophin-releasing hormone stimulation. This suggests that BN may be linked to a complex neuroendocrine dysfunction of the HPA axis [127].

In study [37], rTMS reduced cortisol levels in patients with BN. This is a valuable observation—rTMS can modulate and reduce the activity of the HPA axis. The mechanisms of this action are unclear, but this effect may counteract the negative impact of stress on increased appetite and binge eating, which may result in a reduction of behaviors related to BN—binge eating, purging, and vomiting.

## 6. Limitations and Future Directions

Although rTMS in BN shows therapeutical promise, the low number of robust RCTs, small patient samples, and lack of use of advanced neuroimaging tools to investigate the mechanisms of action, current study findings should be approached with caution. The following describes the limitations of current research and suggests how to conduct future work.

### 6.1. Sample Size and Generalizability

The sample sizes in the included studies were very few, most of them with a small number of subjects, where pilot studies [36,38,41] enrolled only between eight and 22 subjects as an example and case studies [43,44,45] focused on individual cases. Even the larger randomized controlled trials (RCTs) had modest sample sizes, such as 47 participants [35] and 37 participants [40]. Small sample sizes reduce the statistical power of studies and make it more likely that the true effects are not identified (Type II errors). Finally, the small sample sizes will also seriously undercut generalizability of any findings to other individuals with BN. Wider, more diverse samples need to be used in order to generalize the results of this study over different demographic domains (increased age diversity and sex differences, illness severity ranges).

### 6.2. Handedness and Demographics

The handedness of participants was not consistently reported across the included studies, and only a few studies explicitly examined the impact of handedness on rTMS outcomes. For instance, study [38] compared the effects of rTMS on BN in both left-handed and right-handed individuals, finding differential effects that suggest handedness may play a role in treatment response. However, other studies [34,37,39,41,42,43,44,45] did not report the handedness of participants, potentially overlooking a variable that could influence outcomes due to hemispheric differences in brain function and responses to stimulation. Additionally, most studies predominantly included female participants, which limits the generalizability of the findings to male individuals with BN. For example, studies [35] and [40] involved only female participants. This gender imbalance means the results may not fully represent the effects of rTMS across both genders, as male individuals may respond differently to treatment due to hormonal, psychological, and physiological differences. More research is needed to explore the effects of rTMS in diverse demographic groups, including both males and females, different age ranges, and individuals with varying baseline characteristics. This would help in understanding the broader applicability and effectiveness of rTMS for BN across entire affected populations.

### 6.3. Short Follow-Up Periods

Many of the included studies had relatively short follow-up periods, limiting the ability to assess the long-term effects of rTMS on BN symptoms. For instance, studies [35] and [40] evaluated outcomes only within a few weeks post-treatment, focusing primarily on immediate and short-term effects without extended monitoring. In study [43], follow-up assessments were conducted up to 12 weeks post-treatment, which provided some insight into the medium-term effects of rTMS, but longer-term outcomes were not assessed. Study [44] reported a follow-up duration of 64 days post-treatment, noting some recurrence of symptoms after this period. The lack of long-term follow-up data means that the durability of rTMS on BN remains unclear. It is possible that initial improvements could diminish over time, and without extended follow-up, it is difficult to determine the necessity and frequency of maintaining treatments.

### 6.4. Adverse Effects Monitoring

Monitoring and reporting adverse effects were not consistently detailed across the included studies, making it challenging to fully understand the safety profile of rTMS in treating BN. Some studies did report minor adverse effects, such as transient headaches or scalp discomfort, but comprehensive reporting was often lacking. For example, study [42] specifically examined cardiovascular safety and found no significant effects on blood pressure and heart rate, indicating that rTMS could be safe from a cardiovascular perspective. However, other studies did not provide detailed information on potential side effects, such as cognitive changes, mood disturbances or long-term adverse effects. Consistent and thorough monitoring of adverse effects is crucial for ensuring patient safety and optimizing treatment protocols. Future studies should include standardized protocols for adverse effect monitoring and reporting, covering both immediate and long-term side effects. This would help in identifying any potential risks associated with rTMS and in developing strategies to mitigate these risks, thereby improving the overall safety and tolerability of rTMS as a treatment for BN. 

### 6.5. Complete Outcome Data Missing

Study [37] showed a beneficial effect of rTMS on the number of binge eating episodes and provided the number of binge eating episodes before treatment, but did not provide numerical data on the change after treatment. This makes the treatment effect difficult to encapsulate and makes it impossible to perform statistical calculations for further analyses, for example in the form of a meta-analysis. Future studies should be sure to report specific data on treatment outcomes.

### 6.6. Use of Validated Measurement Tools Instead of Diaries

Some studies have measured the occurrence of binge eating episodes and vomiting episodes using diaries. Future studies should use validated measures to measure these symptoms, as they are central to BN.

### 6.7. Avoid Measuring a Given Pathophysiology or Symptom over a Longer Period of Time

Some studies measured the occurrence of, for example, binge eating episodes or vomiting episodes over 15 days. To facilitate analyses of future studies and facilitate comparability, we suggest that we measure the occurrence of symptoms within a single day.

### 6.8. Focus on Core Symptoms of BN in Future Studies

The basic symptoms of BN are binge eating episodes, induced vomiting, and purging behaviors. Surprisingly, only two of the 12 studies included in this review examined the effect of rTMS on the number of vomiting episodes. In the discussion, we cautiously assessed that, despite the small number of studies and rather mixed results, rTMS appears to reduce the incidence of vomiting. More studies have tested the effect of rTMS on the number of binge eating episodes. If future studies are to demonstrate the effectiveness of treating BN with rTMS, they must focus primarily on the effects on the symptoms and pathophysiology of this disorder.

### 6.9. Validation of rTMS Protocols

The included studies used different rTMS protocols. This makes it difficult to draw conclusions regarding the most effective stimulation parameters in BN. The same protocols can be adopted from depression studies because, primarily, a co-morbidity like depression has been commonly associated with eating disorders, particularly BN [128]. Actually, research has shown that eating disorders raise the likelihood of provoking depression, and that depression predisposes to eating disorders [129]. The binge–purge cycle was found to be the driving force behind the association between depression and BN in a study that looked at various theories surrounding the relationship [130]. Depression and adverse effects fluctuate along the cycle; one hypothesis contends that BN causes depression, while another contends that BN is a result of depression [130]. These explanations were later shown to be unsatisfactory, raising questions about the connection between BN and depression. Patients with BN are more likely to attempt suicide if they are depressed [131,132]. Secondly, the use of rTMS for the treatment of depression is the most frequently researched application of brain stimulation with proven effectiveness. This resulted in the FDA officially approving rTMS as a treatment option for depressive disorders. Much research effort has been made to determine the parameters that influence the effectiveness of rTMS in the treatment of depression. The work of Hsu et al. [133] showed that higher pulse/session and total pulses in rTMS protocols may have a positive correlation with a non-depressed outcome during the first two months of treatment, and also that the third and fourth weeks of treatment seemed to be the most effective, and the acute rTMS phase’s positive ascending slope may indicate a dose-response link between the length of stimulation and the reduction of depression. For the 1-week, 2-week, and 3-week stimulation regimes, the highest effectiveness was noted following the conclusion of rTMS treatment. This result demonstrated the characteristic of rTMS’s delayed effects. In the context of the current review, these results show that greater improvements in depression can be achieved when using a higher number of pulses per session, and that it is also worth using 3-week protocols to see noticeable improvements in depression scores. In Hsu et al. [134], six rTMS parameters were examined to determine their impact on treatment-resistant depression. These parameters are pulses per session, treatment duration, total sessions, frequency, total pulses and intensity. Greater stimulation intensity (100%MT up to 120%MT), greater stimulation frequency (10 Hz up to 20 Hz) and an appropriate number of pulses (i.e., total sessions, treatment duration, more pulses per session, total pulses) were found to be related to treatment success. It is therefore worth examining the impact of these parameters on depression in BN to see whether they will result in a greater improvement in the results.

### 6.10. Use of New Measurement Tools (Neuroimaging, Biomarkers)

To understand the mechanisms of action of rTMS in BN, it is necessary to include modern research methods in future studies. First of all, functional magnetic resonance imaging (fMRI) should be used, which will enable the detection of the effect of rTMS on the activity and functional connectivity of the brain. Plain electroencephalography (EEG) and quantitative electroencephalography (QEEG) may also be useful. To investigate the effect on the serotonin system, it is necessary to use positron emission tomography (PET).

Blood biomarkers can also be used. Studies indicate that blood serotonin measurements may be a useful diagnostic tool in patients with BN [135]. Furthermore, it is worth examining the effect of rTMS on the parameters mentioned in this review—BDNF and neuro-inflammation markers.

## 7. Conclusions

Preliminary evidence indicates that the use of rTMS in patients with BN is an effective method of treating the basic symptoms of this disorder—the number of binge eating and vomiting episodes, and food cravings. Additionally, rTMS improves the symptoms of depression and anxiety. Mixed results mainly concern the impact on cognitive functions and the broad pathology of BN. Existing studies have numerous limitations—the low number of patients, the lack of use of standardized tools to measure the most important symptoms of BN, and the lack of neuroimaging and other methods that would allow for understanding the mechanisms of action of rTMS. The potential mechanisms of action we detail are theoretical and require verification. However, this work is the first to demonstrate the potential of rTMS in the treatment of the mysterious disorder bulimia nervosa.

## Figures and Tables

**Table 1:** Studies included in the review.

Study	Sample Size	Study Design	rTMS Parameters	Outcomes Measured	Key Results
[34]	39 (17 real, 22 sham)	Randomized Controlled Trial (RCT)	10 Hz, 110% motor threshold, 20 trains of 5 s with 55 s intervals, left DLPFC, 1000 pulses/session	Inhibitory control (Go/No-Go, BIS), Decision-making (IGT), Sustained attention (D2 Test)	Improved inhibitory control in real rTMS group; No significant change in decision-making; Positive change in sustained attention in both groups
[35]	47 (23 real, 24 sham)	RCT	10 Hz, 110% motor threshold, 20 trains of 5 s with 55 s intervals, left DLPFC, 1000 pulses/session	Binge episodes, Vomiting episodes, Depression (MADRS)	No significant reduction in binge episodes; No significant reduction in vomiting episodes; Marginally significant drop in depression score
[36]	8	Single-group pre-post study	10 Hz, 110% motor threshold, 15 trains of 5 s with 55 s intervals, left DLPFC, 1000 pulses/session	Food craving (FCQ-SS), Anxiety (HADS), Depression (HADS), Eating disorder symptoms (EDI-2, EDEQ), Disease severity (CGI), Functioning (GAF)	Non-significant reduction in eating disorder symptoms; Significant improvement in anxiety and functioning; No significant effect on food craving or disease severity
[37]	22 (11 real, 11 sham)	Double-blind RCT	10 Hz, 110% motor threshold, 20 trains of 5 s with 55 s intervals, left DLPFC, 1000 pulses/session	Food craving (FCQ-S, VAS), Cortisol concentration	Real rTMS group less likely to have binge episodes; Trend towards reducing food cravings; Significantly lower cortisol concentration in real rTMS group
[38]	21 (7 left-handed, 14 right-handed)	Single-group pre-post study	10 Hz, 110% motor threshold, 20 trains of 5 s with 55 s intervals, left DLPFC, 1000 pulses/session	Food craving (FCQ-S), Mood (VAS)	Reduction in food craving (FCQ-S); Worsening of mood in left-handed participants
[39]	14 (7 real, 7 sham)	RCT	20 Hz, 120% motor threshold, 10 trains of 10 s with 60 s intervals, left DLPFC, 2000 pulses/session	Binge episodes, Vomiting episodes, Depression (HDRS, BDI), Obsessive-compulsive symptoms (YBOCS)	Decrease in binge and vomiting episodes in both groups; Improvement in self-rated depression (BDI) and obsessive–compulsive symptoms (YBOCS)
[40]	37 (17 real, 20 sham)	RCT	10 Hz, 110% motor threshold, 20 trains of 5 s with 55 s intervals, left DLPFC, 1000 pulses/session	Food craving (FCQ-S), Urge to eat, Hunger, Mood, Tension (VAS)	Significant reduction in urge to eat in real rTMS group; Improvement in food craving (FCQ-S) in both groups, greater in real rTMS
[41]	33 (15 real, 18 sham)	RCT	10 Hz, 110% motor threshold, 20 trains of 5 s with 55 s intervals, left DLPFC	Selective attention (Stroop Color Word Task)	No improvement in selective attention
[42]	38 (18 real, 20 sham)	RCT	10 Hz, 110% motor threshold, 20 trains of 5 s with 55 s intervals, left DLPFC	Blood pressure, Heart rate	No effect on blood pressure or heart rate
[43]	1	Single case study	25 Hz, 110% motor threshold, 2 s trains with 25 s intervals, left DLPFC	Depression (HDRS), Binge-purge episodes	Significant reduction in depression and binge–purge behaviors; Complete remission by the third week
[44]	1	Single case study	10 Hz, 120% motor threshold, 60 trains of 5 s with 10 s intervals, bilateral DMPFC, 3000 pulses/session	Depression (BDI-II, HDRS), Binge-purge episodes	Complete remission of depression and disordered eating; Relapse after 64 days
[45]	1	Single case study	20 Hz, 80% motor threshold, 10 trains of 10 s with 60 s intervals, left DLPFC	Depression (HDRS, BDI), Binge-purge episodes	50% reduction in depression (HDRS); No binge–purge episodes post-treatment
